# Regulation of Toll-like Receptor 5 Gene Expression and Function on Mucosal Dendritic Cells

**DOI:** 10.1371/journal.pone.0035918

**Published:** 2012-04-24

**Authors:** Ting Feng, Yingzi Cong, Katie Alexander, Charles O. Elson

**Affiliations:** 1 Department of Microbiology, University of Alabama at Birmingham, Birmingham, Alabama, United States of America; 2 Department of Medicine, University of Alabama at Birmingham, Birmingham, Alabama, United States of America; 3 Departments of Microbiology/Immunology and Pathology, University of Texas Medical Branch, Galveston, Texas, United States of America; Ulm University, Germany

## Abstract

Toll-like receptor (TLR) 5 has been shown to maintain intestinal homeostasis and regulate host defense against enterobacterial infection. However, how TLR5 expression is regulated and its function in the intestine have not been fully elucidated. Here we demonstrate that mucosal dendritic cells (DCs), but not splenic DCs, express high levels of TLR5 protein. Alternatively spliced *Tlr5* transcripts were identified but it did not explain the selective expression of TLR5 on mucosal DCs. Treatment with various bacterial ligands downregulated BMDC TLR5 expression, while retinoic acid and host stromal cell-derived signals promoted TLR5 expression in a TGF-β-independent mechanism. Signaling through TLR5 restrained regulatory T (Treg) cell generation, and accordingly, TLR5^−/−^ mice displayed increased frequencies of Foxp3^+^ Treg cells in the intestinal lamina propria. Our data indicate that bacterial and host factors differentially regulate DC TLR5 expression. TLR5 signaling regulates immune responses towards the microbiota via modulation of the Treg/effector T cell balance.

## Introduction

Toll-like receptors (TLRs) recognize a variety of conserved microbial components and are able to induce innate immune responses [1]. They are abundantly expressed on innate cells such as macrophages, and dendritic cells (DCs), and serve as an important link between innate and adaptive immune systems. In the gastrointestinal tract, TLRs are expressed in different combinations by a wide variety of cell types, including intestinal epithelial cells (IECs), subepithelial myofibroblasts and immune cell subsets (e.g. macrophages, DCs, B cells and T cells) within the lamina propria (LP). Interestingly, the expression of TLRs by specific cell types differs under different local environments. For example, bone marrow-derived DCs (BMDCs) express high levels of TLR4, whereas lamina propria CD11c^+^ DCs (LPDCs) do not express TLR4, probably to avoid unwanted inflammatory responses to the exposed lipopolysaccharides (LPS), a TLR4 ligand, in the intestinal lumen. However, LPDCs do express other TLRs, such as TLR2, 5 and 9, and also effectively respond to their ligands [2]–[4]. TLRs can be localized either on the cell surface (TLR1/2/4/5/6) or in intracellular compartments (TLR3/7/8/9). While TLR2 and TLR4 are preferentially localized at the apical pole of differentiated enterocytes, TLR5 is expressed at the basolateral pole of the intestinal epithelium. The expression of TLR2 and TLR4 is substantially increased in primary IECs and LP mononuclear cells throughout the lower gastrointestinal tract in active Crohn's disease and ulcerative colitis. Thus, different TLR expression patterns in different locations under different environments may reflect different functional necessities of TLR-ligand recognition in different strategic locations [5], [6].

Flagellin, the structural element of bacterial flagella, can stimulate innate responses through ligation with TLR5 as well as activating adaptive responses through its antigenic domain [7]–[9]. TLR5 recognizes the conserved domain of the flagellin monomer [10], and is expressed on the basolateral surface of intestinal epithelial cells [2]. TLR5-flagellin ligation has been considered as crucial for the detection of invasive flagellated bacteria at the mucosal surface [11]. Different from humans, murine systemic macrophages and DCs lack TLR5 expression and do not respond to soluble flagellin [12]. However, murine intestinal lamina propria DCs express TLR5 transcripts and are able to respond to flagellin [13], [14]. Flagellin-stimulated CD11c^+^ lamina propria DCs do not produce interleukin (IL)-10 or tumor-necrosis factor (TNF)-α, but produce IL-6 and IL-12 [14], [15]. The unique expression profile of TLRs on CD11c^+^ lamina propria DCs seems to be closely related to the specific microenvironment in the intestine. Although some studies suggest that TLR5 is critical in maintenance of intestinal immune homeostasis and host defense against bacterial infection, how TLR5 gene expression is regulated and its function in the mucosa have not been fully elucidated.

In this report, we investigated how TLR5 gene expression on mucosal DCs is regulated and the role of TLR5 signaling in adaptive immune responses. We demonstrate that intestinal lamina propria DCs express TLR5 protein to a much greater extent than do spleen DCs. The *Tlr5* transcript was identically spliced in intestinal and splenic DCs, and thus did not explain the preferential expression of TLR5 in the colon. Microbial TLR ligands downregulated, while retinoic acid (RA) and host intestinal stromal cell-derived factors synergistically promoted DC TLR5 expression. Functionally, signaling through TLR5 restrained regulatory T (Treg) cell generation, but promoted effector T cell responses.

## Results

### Lamina propria DCs, but not splenic DCs, express high levels of TLR5 protein

**Figure 1:**
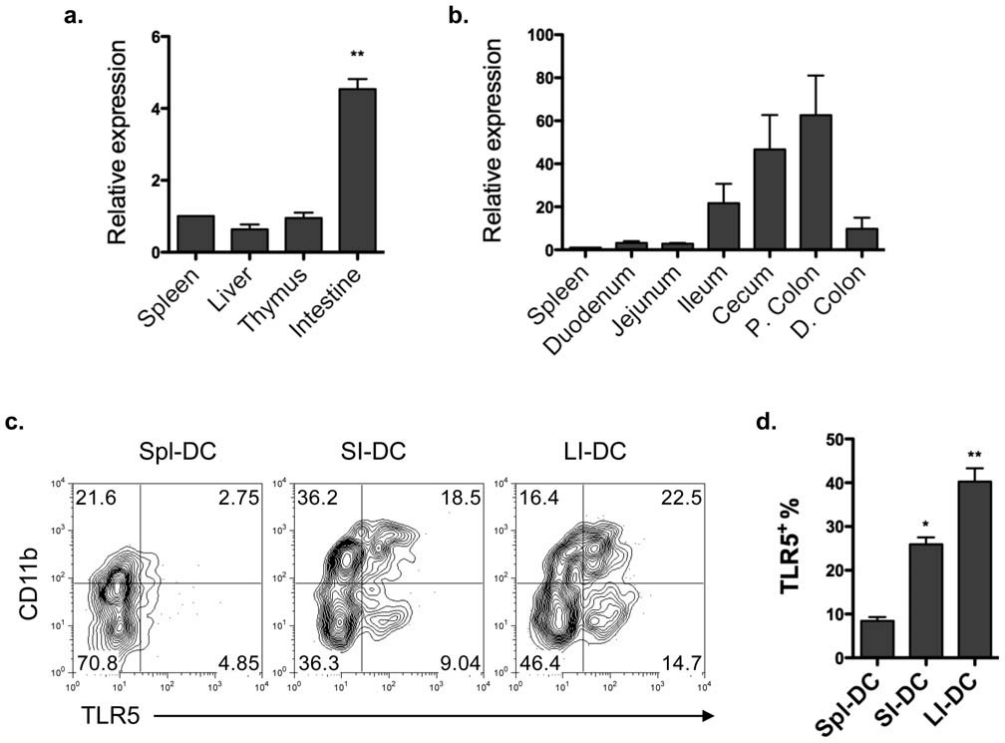
Lamina propria DCs, but not splenic DCs, express high levels of TLR5 protein. (**a**) Quantitative real-time PCR of *Tlr5* expression in the spleen, liver, thymus and intestine homogenates of C57BL/6 mice normalized to *18S* mRNA expression. **P<0.01; compared to spleen. (**b**) Quantitative real-time PCR of *Tlr5* expression in different intestinal fragments, including duodenum, jejunum, ileum, cecum, proximal colon (P. colon) and distal colon (D. colon), as compared to spleen *Tlr5* expression. (**c**) TLR5 expression on splenic DCs (Spl-DC), small intestinal DCs (SI-DC) and large intestinal DCs (LI-DC) from pooled three mice was determined by flow cytometry. Plot numbers represent the percentage of CD11c^+^ cells in the respective quadrants. (**d**) Aggregate data of percentages of TLR5^+^ cells among CD11c^+^ cells. *P<0.05; **P<0.01; compared to splenic DCs. Data are aggregate (a, b, d; mean ± SEM) or representative (c) of three experiments, 2–3 mice for each experiment.

We first examined the gene expression of murine *Tlr5* among different tissues including the spleen, liver, thymus and intestine by quantitative real-time PCR. *Tlr5* mRNA was found to be highly expressed only in the intestine (Figure 1a). To determine the *Tlr5* gene expression pattern along the whole intestinal tract, different segments of the intestine were obtained and assessed for *Tlr5* expression by quantitative real-time PCR. We found that *Tlr5* gene was selectively expressed at a higher level in the ileum and highest in the cecum and proximal colon than the rest of the intestinal tract (Figure 1b). It has been reported that *Tlr5* mRNA is highly expressed on intestinal lamina propria CD11c^+^CD11b^+^ DCs, but not on splenic DCs [13], [14]. We next examined TLR5 protein expression on mucosal DCs. Small and large intestinal lamina propria DCs and splenic DCs were stained with anti-TLR5 antibody and analyzed by flow cytometry. Consistent with previous reports, more lamina propria DCs expressed TLR5 than did splenic DCs, in that approximately 7.6% of CD11c^+^ DCs expressed TLR5 in the spleen, whereas 37.2% of CD11c^+^ DCs expressed TLR5 in the large intestine (Figure 1c and d). Several subsets of DCs are present in the intestinal lamina propria, functioning differently to defend against pathogenic microorganisms and maintain intestinal immune homeostasis [16]–[19]. We then determined if there is a differential TLR5 expression pattern among different subsets of lamina propria DCs. CD11c^+^CD11b^+^ lamina propria DCs predominantly expressed TLR5. However, in contrast to previous reports that only the CD11c^+^CD11b^+^ subset expresses TLR5 [13], [14], a fraction of CD11c^+^CD11b^−^ lamina propria DCs also expressed TLR5 (Figure 1c). Our data that TLR5 was expressed most on colon lamina propria DCs suggested that local intestinal environmental factors may play a role in regulating TLR5 expression and hence may affect functions of TLR5-expressing mucosal DCs.

### Microbial TLR ligands downregulate DC TLR5 expression

**Figure 2:**
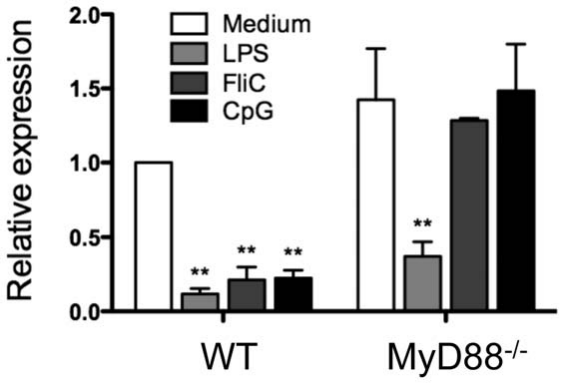
TLR ligand stimulation downregulates DC TLR5 expression. BMDCs from wild type and MyD88^−/−^ mice were stimulated with LPS (1 µg/ml), FliC (10 ng/ml) or CpG (3 µg/ml) for 4 h. TLR5 expression was analyzed by real-time PCR and normalized to *Gapdh* mRNA expression. Bar chart represents aggregate data with mean ± SEM of three experiments. **P<0.01; compared to untreated wild type BMDCs. One wild type and one MyD88^−/−^ mouse for each experiment.

The intestinal tract is colonized with an abundant microbiota and it has been shown that TLR4 expression on DCs can be enhanced by stimulation with its ligand LPS [20], [21]. We asked whether flagellin stimulation is responsible for the high levels of TLR5 expression on lamina propria DCs. Bone marrow-derived DCs (BMDCs) were treated with various TLR ligands, including LPS (TLR4 ligand), flagellin (TLR5 ligand) and CpG ODN (TLR9 ligand), and TLR5 expression was assessed by real-time PCR 4 h later. Unexpectedly, TLR5 mRNA expression was downregulated by TLR ligand stimulation (Figure 2). The majority of TLR signaling pathways are dependent on the adapter molecule MyD88 [22], [23]. To determine whether the downregulation of TLR5 by TLR ligand stimulation is through the MyD88 pathway, BMDCs from MyD88^−/−^ mice were similarly treated with LPS, flagellin, and CpG. As shown in Figure 2, MyD88 deficiency abrogated flagellin- and CpG-induced downregulation of TLR5, but had no effect on the LPS-induced decrease of TLR5 expression, the latter of which was possibly due to signaling through the MyD88-independent pathway. Together, these data demonstrate that direct stimulation by commensal bacteria and their TLR ligands are not responsible for high TLR5 expression on DCs.

### Determination of TLR5 transcription start sites and alternative transcripts

**Figure 3:**
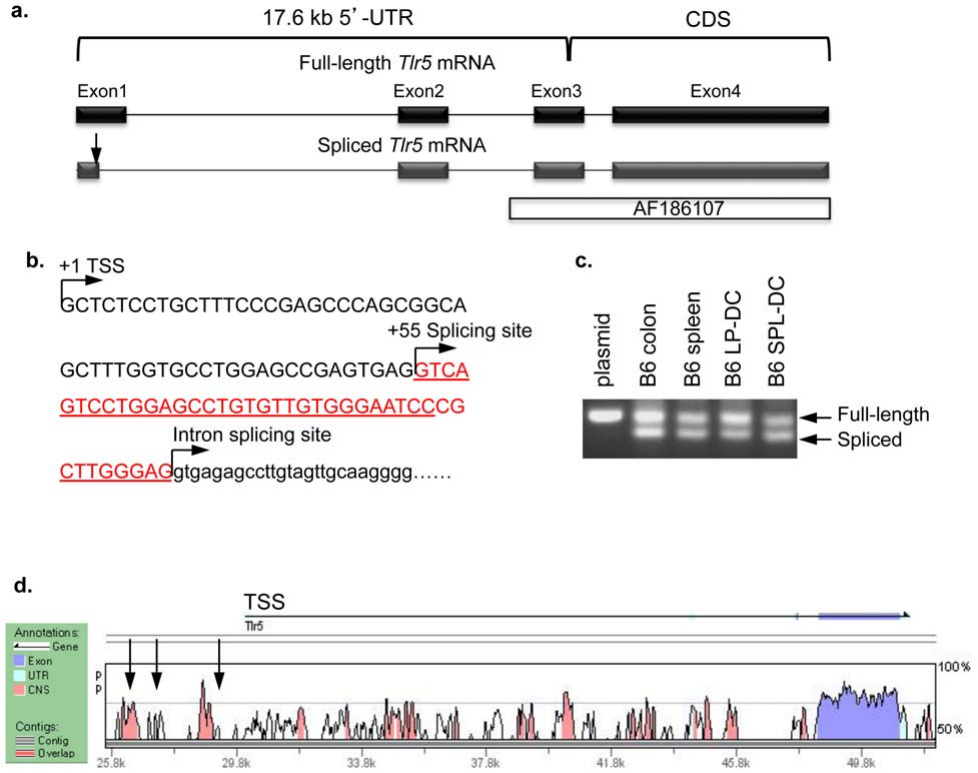
Determination of TLR5 transcription start sites and alternative transcripts. (**a**) A schematic diagram of the chromosomal region encompassing the RLM-RACE-identified mouse *Tlr5* gene transcription start site (TSS), exons 1–4 and introns 1–3. An alternative-splicing variant (indicated by the arrow in exon 1), as well as the GenBank sequence AF186107 are also shown. (**b**) Exon 1 sequence (upper case) and partial intron sequence flanking exon 1 and 2 (lower case) with indication of alternative splicing site (+55). The alternatively spliced exon 1 sequence is underlined. (**c**) PCR analysis of spliced variants in full-length plasmid control, colon or spleen tissue homogenates, and lamina propria or splenic DCs. (**d**) VISTA analysis of conserved noncoding sequences (CNS) in the *Tlr5* locus. Mouse sequence is shown on the x-axis and percentage similarity to human on the y-axis. A schematic representation of the mouse *Tlr5* locus with 5′- and 3′-UTRs is shown, with the horizontal arrow indicating direction of transcription denotes position of the *Tlr5* gene. Peaks show CNS defined as longer than 100 bp and having greater than 70% sequence identify. Vertical arrows indicate MatInspector-predicted putative RAR-responsive elements (−827 to −851, −2267 to −2292, and −3755 to −3779). UTR, untranslated region; CDS, coding sequence; TSS, transcription start site. The result shown was one representative of three independent experiments of RNA preparation. For each experiment, DCs were FACS-sorted from a pool of 4–5 mice.

As a first step to study gene regulation of murine TLR5, we determined the transcription start sites (TSS) of *Tlr5* transcripts in intestinal DCs by RNA ligase-mediated rapid amplification of cDNA ends (RLM-RACE), which selectively amplifies capped, full-length transcripts. RLM-RACE identified the 5′-TSS, which was 17.6 kb upstream of the coding region on the mouse genome (Figure 3a). Consistent with the GenBank sequence (NM_016928.2), the transcript was spliced into four exons of a final length of 3211 bp. Our data conflicts with the previous *Tlr5* gene studies which reported a 4286 bp transcript with at least 998 nucleotides of additional 5′-untranslated region (UTR) sequence (AF186107) [24]. When we aligned the deposited GenBank sequence (AF186107) with the whole mouse genome, the coding sequence appeared to be a linear transcript starting within intron 2–3 (Figure 3a). In addition, RLM-RACE revealed an alternatively spliced transcript lacking 41 nucleotides of 3′ exon 1 (Figure 3a and b). Alternative splicing has been demonstrated in other TLRs such as human TLR1, 2, 3, 4, 9 and mouse TLR4, and gene studies suggest preferential expression of certain splice variants in specific tissues [25]. To investigate whether alternative splicing would explain the high expression of TLR5 in intestinal DCs but not in splenic DCs, we analyzed the presence of splicing variants in the colon, spleen, intestinal lamina propria DCs and splenic DCs. However, the *Tlr5* transcript was identically spliced in intestinal and splenic DCs (Figure 3c), and thus did not explain the preferential expression of TLR5 in the intestine.

To identify conserved noncoding sequence (CNS) elements that might regulate *Tlr5* gene expression, we compared approximately 25 kb of DNA sequence encompassing the *Tlr5* locus between human and mouse by using the web-based alignment tool VISTA. As shown in Figure 3d, the coding sequence of the *Tlr5* gene is quite conserved as are a number of noncoding sequences (CNS: greater than 70% homology and greater than 100 bp long). We then searched for putative transcription factor binding sites around 4 kb upstream of *Tlr5* TSS using the web-based program MatInspector, which revealed several RA receptor (RAR) binding sites (Figure 3d). RA is enriched in the intestine, and has profound effects on DCs and T cells in the mucosa [17], [26]. We next asked whether RA might selectively induce TLR5 expression in the intestine.

### RA and host stromal cell-derived factors synergistically enhance DC TLR5 expression

**Figure 4:**
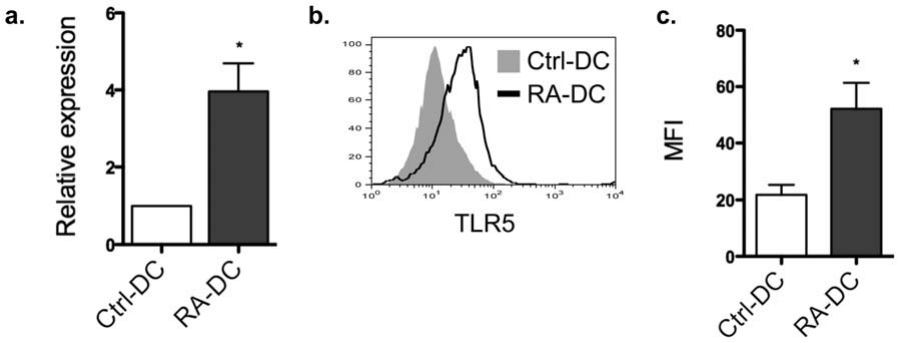
RA upregulates DC TLR5 expression. BMDCs were generated in the absence (Ctrl-DC) or presence of 1 µM RA (RA-DC) from day 3 of an 8-day culture. Control DC and RA-DC TLR5 expression was determined by quantitative real-time PCR (**a**) and by flow cytometry shown as histogram (**b**; shaded histogram, control DCs; solid line, RA-DCs) or as aggregate data (**c**). Bar charts represent aggregate data with mean ± SEM of three experiments (a, c; *P<0.05; compared to control DC). Flow cytometry data (b) is representative of three individual experiments. One wild type mouse for each experiment.

As shown previously, RA, a metabolite of vitamin A, instructs mucosal DC development both phenotypically and functionally [26]. To determine the effect of RA on DC TLR5 expression, RA was added from day 3 of BMDC culture (RA-DCs) in the presence of recombinant GM-CSF. TLR5 expression was determined by quantitative real-time PCR and flow cytometry on day 8 of culture. As shown in Figure 4a, TLR5 mRNA expression by RA-DCs was about 4-fold higher than that of control BMDCs. This was confirmed by flow cytometric analysis of TLR5 expression (Figure 4b and c).

**Figure 5:**
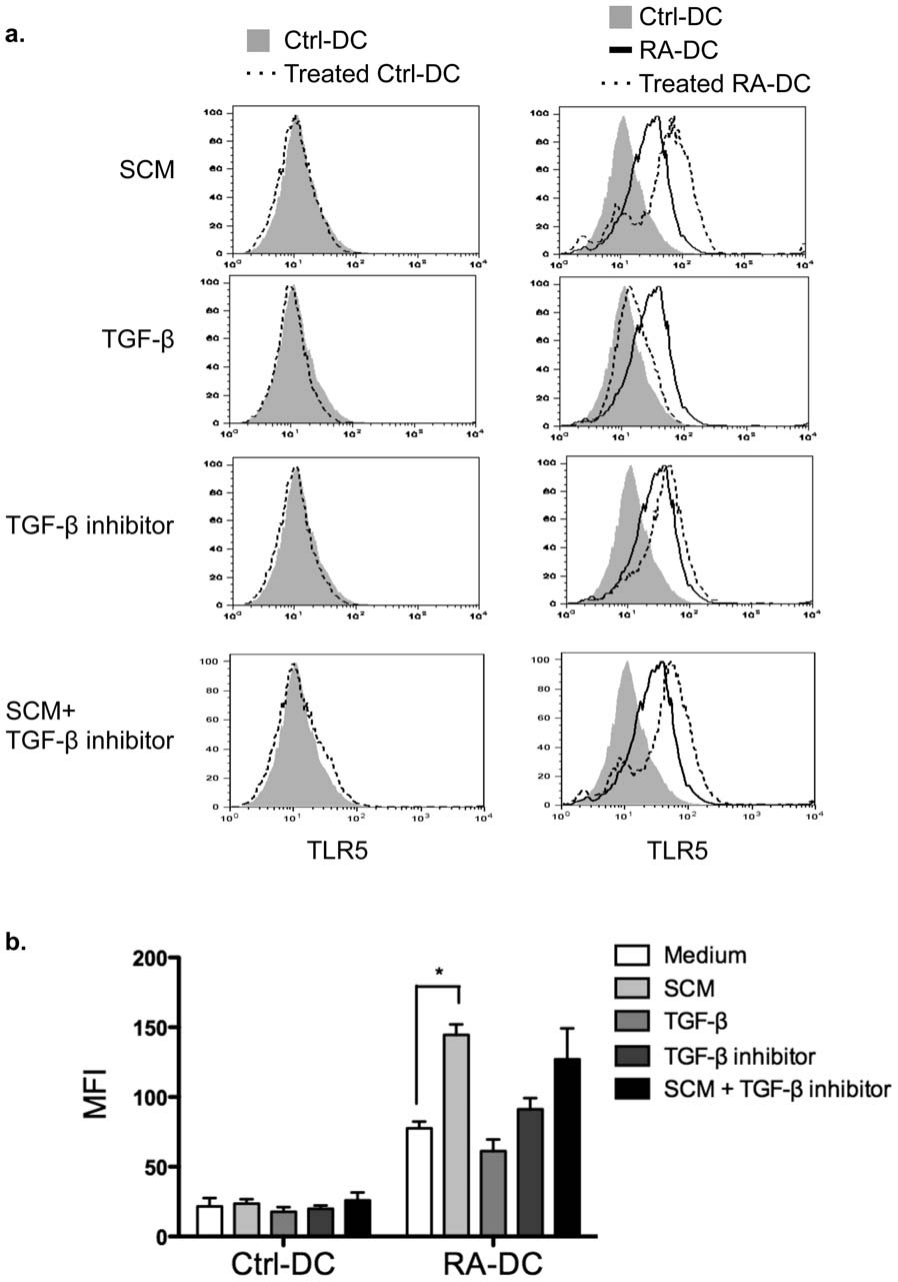
Host stromal cell-derived factors synergistically enhance RA-DC TLR5 expression. Control DCs and RA-DCs were treated with intestinal stromal cell-conditioned medium (SCM 250 µg/ml), TGF-β (10 ng/ml), TGF-β inhibitor (SB-505124 1 µM) or SCM plus TGF-β inhibitor for 24 h, and TLR5 expression was determined by flow cytometry (**a**). Shaded histogram, control DCs; solid line, RA-DCs; dotted line, treated control DCs (left) or RA-DCs (right). Data are representative of three experiments. (**b**) Aggregate data of DC TLR5 MFI with mean ± SEM of three experiments. *P<0.05; compared to untreated DCs. One wild type mouse for each experiment.

It has also been shown that intestinal stromal cell products play a critical role in shaping intestinal macrophage phenotype and cytokine profile [27]. To determine whether stromal cell products also affect TLR5 expression on DCs, control DCs and RA-DCs were treated with stromal cell-conditioned medium (SCM) at 250 µg/ml and TLR5 expression was determined by flow cytometry 24 h later. As shown in Figure 5a and b, treatment with SCM synergistically increased TLR5 expression in RA-DCs as represented by increase of mean-fluorescent intensity (MFI), but not in control DCs. TGF-β appears to be a key stromal cell product that inhibits monocyte cytokine production [27]. We next examined the effect of TGF-β on DC TLR5 expression. Control DCs and RA-DCs were cultured in the absence or presence of TGF-β or the TGF-β inhibitor SB-505124. Although not statistically significant, blockade of TGF-β exhibited the trend to upregulate TLR5 expression on RA-DCs, and addition of TGF-β tended to decrease RA-DC and SCM-treated RA-DC TLR5 expression MFI (Figure 5b), which indicate that stromal cell products enhance RA-DC TLR5 expression through a TGF-β-independent pathway. Taken together, these findings indicate an important role for RA as well as host stromal cell-derived factors in the regulation of TLR5 expression on lamina propria DCs.

### TLR5 ligation downregulates RA-DC and lamina propria DC induction of Foxp3^+^ Treg cells

**Figure 6:**
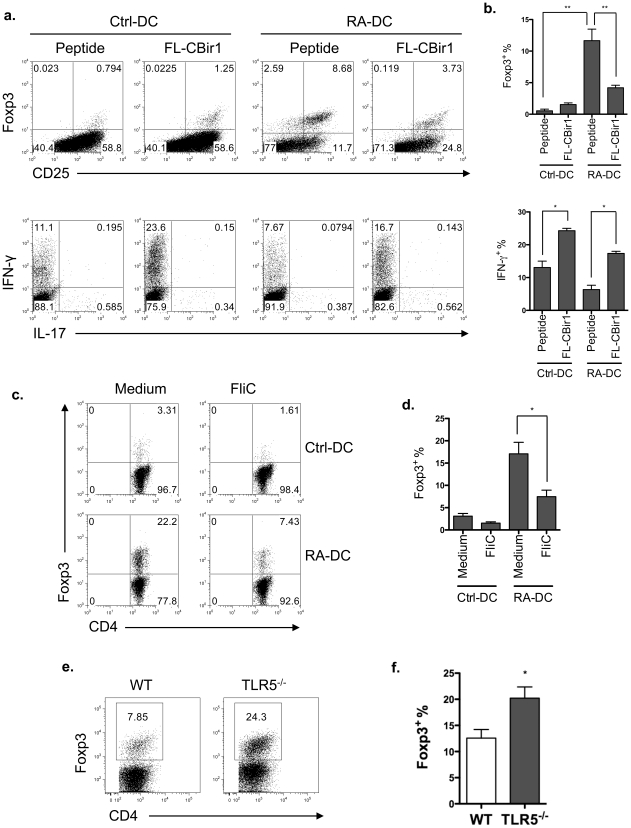
TLR5 ligation inhibits Foxp3^+^ Treg cell generation. (**a**) 0.2×10^5^ CD4^+^ T cells from CBir1-Tg mice were cultured with control DCs or RA-DCs that were pulsed with full-length CBir1 flagellin (FL-CBir1) or CBir1 peptide. Five days later, T cell Foxp3 and cytokine expression were analyzed by intracellular staining. Plot numbers represent the percentage of CD4^+^ cells in the respective quadrants. (**b**) Aggregate data of Foxp3^+^ and IFN-γ^+^ T cells among total CD4^+^ T cells with mean ± SEM from three independent experiments. **P<0.01, *P<0.05 (**c**) CD4^+^ T cells from CBir1-Tg mice were cultured with CBir1 peptide pulsed-control DCs or RA-DCs in the presence of *Salmonella* flagellin FliC for 5 days. CD4^+^ T cell Foxp3 expression was analyzed by intracellular staining. (**d**) Aggregate data of Foxp3^+^ T cells among total CD4^+^ T cells with mean ± SEM from three experiments. *P<0.05 (**e**) Foxp3 expression of the lamina propria T cells from wild type and TLR5^−/−^ mice was determined by flow cytometry. (**f**) Aggregate data of Foxp3^+^ T cells among total CD4^+^ T cells with mean ± SEM from three experiments. *P<0.05. Flow cytometry data (a–c) are representative of at least three experiments. For Figure 6a–d, bone marrow cells were isolated from one wild type mouse; naïve CD4 T cells were isolated from one CBir1-Tg mouse. For Figure 6e–f, lamina propria T cells were isolated from a pool of 2–3 wild type or TLR5^−/−^ mice, respectively.

Flagellin-stimulated CD11c^+^ lamina propria DCs, which do not produce IL-10 but produce IL-6 and IL-12 [13], [14], have been shown to inhibit Treg cells but promote effector T cell responses [28]–[30]. We then investigated whether activation of TLR5 signaling can inhibit mucosal DC induction of Treg cells. We recently generated a TCR transgenic (CBir1-Tg) mouse line specific for the immunodominant microbiota antigen, CBir1 flagellin [31]. Previous data has shown that RA-DCs, similar to lamina propria DCs, are able to induce Treg cells [26]. Naïve CD4^+^ T cells from CBir1-Tg mice were cultured with RA-DCs that were pulsed with full-length CBir1 flagellin (FL-CBir1), which can activate TLR5 signaling, or with CBir1 peptide, which does not bind to TLR5. Five days later, Foxp3 expression was determined by flow cytometry. When cultured with FL-CBir1-pulsed RA-DCs, CBir1-Tg CD4^+^ T cells expressed lower frequencies of Foxp3 and produced higher amounts of IFN-γ than those cultured with CBir1 peptide-pulsed RA-DCs (Figure 6a and 6b). To examine whether TLR5 activation by other flagellin works the same as FL-CBir1, CBir1-Tg T cells were cultured with CBir1 peptide-pulsed RA-DCs in the absence or presence of *Salmonella* flagellin FliC. As shown in Figure 6c and 6d, RA-DC induction of Foxp3 was decreased by the addition of FliC. Lastly, in order to determine whether TLR5 signaling limits Treg cell generation *in vivo*, wild type and TLR5^−/−^ lamina propria T cell Foxp3 expression was analyzed by flow cytometry. As shown in Figure 6e and 6f, lamina propria T cell Foxp3 frequency was significantly higher in TLR5^−/−^ mice than wild type mice. Collectively, our data indicate that signaling through TLR5 inhibits mucosal DC induction of Treg cells, while promoting effector T cells.

## Discussion

The gastrointestinal tract is home to a large microbiota, numbering some 10^14^ bacteria, with the highest microbial density present in the colon [32]. There are species ranging from symbiotic microorganisms to opportunistic pathogens [33]. Symbiotic bacteria benefit the host by supplying essential dietary nutrients, preventing colonization by pathogens and contributing to the development of immune system [34]. The microbiota can also be harmful to the host if the immune response to it is not properly regulated [35]. Thus, the intestinal mucosal immune system has the great challenge of detecting and eliminating true pathogens, keeping opportunistic pathogens in check, and doing so without causing any collateral damage to beneficial microbes or host tissues. The host immune system interacts with the microbiota through both innate and adaptive immune components; however, the mechanisms involved are not completely understood. A previous study has demonstrated that commensal DNA limits Treg cell conversion from naïve T cells and promotes effector T cell function through activation of TLR9 signaling [3]. In the present study, we show that TLR5 signaling in the lamina propria is important in the homeostasis between microbiota and the host immune system via modulation of effector T cell and Treg cell balance.

TLRs expressed on epithelial cells and innate cells (particularly DCs) mediate the dynamic interactions between microbiota and host innate immunity by recognition of a large array of conserved microbial components [1]. Among the TLR family, TLR5 plays an important role in the detection of invasive flagellated bacteria such as *Salmonella typhimurium*, and can protect the host against a variety of insults [15], [36]. Bacterial flagellin, through interaction with TLR5, has been shown to be involved in protection against microbial challenges, chemical injuries, as well as radiation in a TLR5- and MyD88-dependent manner [36], [37]. However, the fundamental question of how TLR5 gene expression is regulated has not been addressed. Previous studies have reported that *Tlr5* transcripts are selectively expressed in the intestine predominantly by CD11c^+^CD11b^+^ DCs [13], [14]. In line with the previous studies, our data further show that *Tlr5* mRNA is preferentially transcribed in the intestine, especially in the large intestine, which expression positively correlates with the microbiota density. However we found that not only CD11c^+^CD11b^+^ but also CD11c^+^CD11b^−^ lamina propria DCs had higher frequencies of TLR5^+^ cells than did their splenic counterparts. It seems plausible that bacterial ligands might stimulate their own TLR expression, thus amplifying TLR signals as a host defense mechanism against invading microorganisms. Nevertheless, we found that direct stimulation by microbial TLR ligands was not responsible for the high levels of TLR5 on DCs.

We next attempted to understand the molecular regulation of the *Tlr5* gene. RLM-RACE identified the transcription start site and an alternative-splicing variant lacking a portion of exon 1. Various other TLR family members such human TLR1, 2, 3, 4, 9 and mouse TLR4 demonstrate alternative splicing in specific tissues exerting negative and positive regulatory functions [25]. However, the *Tlr5* transcript was identically spliced in intestinal and splenic DCs, and thus did not explain the preferential expression of TLR5 in the intestine, perhaps because the alternative splicing site is located within the 5′-UTR and hence does not affect the protein coding sequence (CDS). Although these data cannot completely rule out the possibility that alternative splicing might contribute to the differential expression of TLR5, these data demonstrated at least that alternative splicing does not play a dominant role in this effort. More genetic and functional studies regarding this alternatively spliced transcript will be needed in order to comprehensively appreciate the transcriptional regulation of *Tlr5* gene expression. It could also indicate that other factors, including local environmental factors, regulate TLR5 expression in the intestine.

In our analysis of putative transcription binding sites at the *Tlr5* gene locus, several RAR-responsive elements were identified. Vitamin A, as an essential dietary nutrient, is enriched in the intestinal mucosa. RA, the active metabolite of vitamin A, can be metabolized by retinal dehydrogenases, such as aldehyde dehydrogenase family 1, subfamily A1 and A2 (ALDH1a1 and ALDH1a2). Mucosal DCs express higher levels of *Aldh1a2* than their splenic counterparts, and are able to convert vitamin A into RA *in vitro* [19]. RA educates mucosal DC development [26], and in the present study, we found RA promoted BMDC *Tlr5* gene and protein expression. Of note, not all BMDCs express TLR5 during their differentiation into mucosal-like DCs under the culture with RA. One plausible interpretation is that only precursors of certain types of DCs in bone marrow will develop into TLR5-expressing mucosal-like DCs, as not all LPDCs express TLR5 in the intestine. Intestinal stromal cell products have been reported to shape intestinal macrophage phenotype and function [27]. The presence of TGF-β in the media of cultured stroma and jejunal explants suggests that epithelial and mast cell-released TGF-β binds to the lamina propria extracellular matrix and induces the differentiation of proinflammatory monocytes into non-inflammatory intestinal macrophages [27]. Treatment with stromal factors synergistically increased TLR5 expression in RA-DCs, but not in control DCs, independently of TGF-β. Therefore, differential regulation of TLR5 expression by bacterial TLR ligands and host-derived factors such as RA and stromal cell products, all of which are enriched in the intestinal mucosa, appears to keep DC TLR5 expression in balance under steady-state conditions.

Functionally, it has been shown that TLR5^+^ lamina propria DCs induce differentiation of naïve B cells into immunoglobulin A-producing plasma cells by a mechanism independent of gut-associated lymphoid tissue, and promote development of antigen-specific T helper (Th) 1 and Th17 cells [13]. A more recent study has also defined a role of TLR5 for activation of CD3^−^CD127^+^ immune cells and production of IL-17 and IL-22 in the early defenses against pathogen invasion of host tissues [38]. In our current study, TLR5^−/−^ mice had higher levels of Foxp3^+^ Treg cells than wild type mice. In line with this, TLR5 engagement limited Treg cell generation, but promoted effector T cells *in vitro*. Therefore, high expression levels of TLR5 on lamina propria DCs ensure TLR5^+^ DCs a crucial role in the induction of effector T cell responses against invading flagellated pathogens, while TLR5^−^ DCs may be responsible for maintenance of intestinal homeostasis, likely through induction of Treg cells.

Bacterial flagellin is a dominant target of the elevated humoral antibody response to the commensal microflora that has been associated with Crohn's disease [39], [40]. Our previous study using serologic expression cloning found that 25% of the microbiota antigens targeted by colitic mice were commensal bacterial flagellins [39]. Accumulating evidence suggests that flagellin's immunodominance as an antigen results from its ability to activate innate immunity through ligation with membrane-bound TLR5 or with intracellular receptor Ipaf. Flagellin from *H. pylori*, which does not activate TLR5, fails to elicit any antibody response. However, when administered along with a potent innate adjuvant, it is as immunogenic as *Salmonella* flagellin that is able to activate TLR5 [41]. Flagellin can act as an adjuvant to promote both T cell and humoral antibody responses towards co-administered antigens via interactions with TLR5 [11], [41]–[43]. Direct activation of TLR5^+^CD11c^+^ cells is necessary for the adjuvant activity of flagellin [44]. Thus, the TLR5-mediated innate immune response in the gut has profound influences on adaptive immunity not only to itself but also to other antigens of the microbiota.

In conclusion, we demonstrate in this report that lamina propria DCs express high levels of TLR5. Extrinsic microbial products and intrinsic host cell-derived factors differentially regulate DC TLR5 expression. Signaling DCs via TLR5 inhibits Treg cell differentiation and enhances effector T cell responses. Thus, high levels of TLR5 on lamina propria DCs make TLR5 signaling an important regulator in host immune responses toward flagellated microbes as well as in maintenance of intestinal homeostasis.

## Methods

### Mice

C57BL/6 (B6) mice were purchased from The Jackson Laboratory. B6.CBir1 TCR transgenic (CBir1-Tg) mice [31] were generated and bred in the Animal Facility at the University of Alabama at Birmingham. B6.*MyD88^−/−^* (MyD88^−/−^) mice were kindly provided by Dr. Suzanne Michalek (University of Alabama at Birmingham). B6.*Tlr5^−/−^* (TLR5^−/−^) mice were kindly provided by Dr. Richard Flavell (Yale University). All experiments were reviewed and approved by the Institutional Animal Care and Use Committee of the University of Alabama at Birmingham.

### Antibodies and reagents

Anti-mouse CD4 (RM4-5), CD11b (M1/70), CD11c (HL3), CD25 (PC61), IL-17A (TC11-18H10), and IFN-γ (XMG1.2) were purchased from BD Biosciences. Anti-mouse Foxp3 (FJK-16s) and intracellular staining kit were purchased from eBioscience. Live/Dead Fixable stain was purchased from Invitrogen. Anti-mouse TLR5 (85B152.5) was purchased from Novus Biologicals. All-trans RA and TGF-β inhibitor (SB-505124) were purchased from Sigma-Aldrich. Recombinant human TGF-β was purchased from R&D Systems.

### Quantitative Real-Time PCR

Total RNA was extracted with TriZol reagent and followed by cDNA synthesis with Superscript reverse transcriptase (Invitrogen). Quantitative PCR reactions were performed using TaqMan® Gene Expression Assays for *Tlr5* and *Gapdh* or *18S* (Appliedbiosystems) on Bio-Rad iCycler (Bio-Rad) and all data were normalized to *Gapdh* or *18S* mRNA expression.

### Isolation of spleen and lamina propria DCs

Spleen and lamina propria leukocytes were isolated as previously described [45]. Briefly, small and large intestines were removed and sliced. After removing epithelium by gentle shaking in Ca^2+^/Mg^2+^-free HBSS supplemented with 1 mM EDTA and 2% FBS for 40 min at 37°C, the tissues were resuspended in digestion medium containing RPMI 1640, 5% FBS, 0.5 mg/ml collagenase type IV, and incubated for 40 min at 37°C by gentle shaking. The cells were passed through a mesh, then centrifuged and the pellet was resuspended in 40% Percoll and carefully overlaid onto 70% Percoll. The interface containing the lamina propria leukocytes was collected. Leukocytes from spleen and lamina propria were further isolated by magnetic sorting (Miltenyi Biotec) or by FACS sorter.

### Generation and activation of BMDCs

Bone marrow cells were isolated as described previously [46]. Briefly, bone marrow cells were suspended at 2.5×10^5^/ml in complete RPMI 1640 media containing 10% heat-inactivated FCS (Atlanta Biologicals), 25 mM HEPES buffer, 2 mM sodium pyruvate, 50 mM 2-mercaptoethanol, 100 IU/ml Penicillin, and 100 µg/ml Streptomycin (Cellgro Mediatech). The cells were cultured in the presence of 20 ng/ml GM-CSF (R&D Systems) in 6-well plates at 37°C in 5% CO_2_ in humid air. One µM RA was added from day 3 of an 8-day culture. On day 8, BMDCs were harvested and plated at 1×10^6^/ml per well in 24-well plates in the presence of recombinant full-length CBir1 flagellin [39], *Salmonella* flagellin FliC (InvivoGen) LPS (Sigma-Aldrich), or CpG ODN (InvivoGen) for 4–6 hours.

### 5′-RNA ligase-mediated RACE (RLM-RACE)

Transcription start sites were determined by using total RNA isolated from FACS-sorted mouse intestinal DCs for cDNA synthesis with the FirstChoice RLM-RACE kit (Ambion). The following TLR5-specific primers were used to amplify endogenous full-length 5′-cDNA fragments of mouse TLR5. First-reaction primer: CAGGGAATCTGGGTGAGGTTACAG. Nested primer: CTGGCCATGAAGATCACACCTATG. PCR products were cloned into pCR2.1-TOPO (TOPO Cloning kit; Invitrogen) and inserts from ten individual plasmid-containing bacterial colonies were reamplified by PCR and directly sequenced. The following primers were used to detect splicing variants. First-reaction primer set: forward CGAGCCCAGCGGCAGCTT and reverse CAGGGAATCTGGGTGAGGTTACAG. Nested primer set: forward TGGTGCCTGGAGCCGAGT and reverse CTGGCCATGAAGATCACACCTATG.

### Stromal cell-conditioned medium (SCM)

Stromal cell-conditioned medium (SCM) was prepared as previously reported [27]. Briefly, cell-depleted lamina propria stroma (1 g wet weight stromal tissue per milliliter) was cultured in RPMI for 24 hours. Lamina propria SCM was harvested, sterile filtered (0.2-µm Syringe Filter; Corning Inc.), and frozen at −70°C. Endotoxin, protein, and protease content were determined by commercially available ELISA assays.

### CD4^+^ T cell isolation

CD4^+^ T cells were isolated as previously described [47] by using anti-mouse CD4-magnetic beads (BD Biosciences) following the manufacturer's instructions.

### Statistical analysis

Levels of significance were determined by Student's *t* test. P values of <0.05 were considered to be statistically significant.
